# Candidate genes underlying the quantitative trait loci for root-knot nematode resistance in a *Cucumis hystrix* introgression line of cucumber based on population sequencing

**DOI:** 10.1007/s10265-019-01147-1

**Published:** 2019-10-25

**Authors:** Chunyan Cheng, Xing Wang, Xuejiao Liu, Shuqiong Yang, Xiaqing Yu, Chuntao Qian, Ji Li, Qunfeng Lou, Jinfeng Chen

**Affiliations:** College of Horticulture, Nanjing Agricultural University, No. 1 Weigang Street, Nanjing, 210095 China

**Keywords:** Cucumber, Root-knot nematode, QTL, SNP, Bin map

## Abstract

The southern root-knot nematode (RKN), *Meloidogyne incognita* (Kofoid & White) Chitwood, is one of most destructive species of plant parasitic nematodes, causing significant economic losses to numerous crops including cucumber (*Cucumis sativus* L. 2n = 14). No commercial cultivar is currently available with resistance to RKN, severely hindering the genetic improvement of RKN resistance in cucumber. An introgression line, IL10-1, derived from the interspecific hybridization between the wild species *Cucumis hystrix* Chakr. (2n = 24, HH) and cucumber, was identified with resistance to RKN. In this study, an ultrahigh-density genetic linkage bin-map, composed of high-quality single-nucleotide polymorphisms (SNPs), was constructed based on low-coverage sequences of the F_2:6_ recombinant inbred lines derived from the cross between inbred line IL10-1 and cultivar ‘Beijingjietou’ CC3 (hereinafter referred to as CC3). Three QTLs were identified accounting for 13.36% (qRKN1-1), 9.07% and 9.58% (qRKN5-1 and qRKN5-2) of the resistance variation, respectively. Finally, four genes with nonsynonymous SNPs from chromosome 5 were speculated to be the candidate RKN-resistant related genes, with annotation involved in disease resistance. Though several gaps still exist on the bin-map, our results could potentially be used in breeding programs and establish an understanding of the associated mechanisms underlying RKN resistance in cucumber.

**Electronic supplementary material:**

The online version of this article (10.1007/s10265-019-01147-1) contains supplementary material, which is available to authorized users.

## Introduction

The southern root-knot nematode (RKN), *Meloidogyne incognita* (Kofoid & White) Chitwood, can cause severe yield loss of many crop species, including cucumber (*Cucumis sativus* L.), in China and around the world (Fassuliotis 1970; Ma et al. 2014; Milligan et al. 1998; Pham et al. 2013; Shukla et al. 2017; Xu et al. 2013). Although chemicals, especially nematicides, have been applied successfully in controlling nematodes in many crops for decades, the ecologically unfriendly ingredients including 1,2-dibromo-3-chloropropane (DBCP) and ethylene dibromide (EDB) made nematicides less effective and placed heavy emphasis on the development and the usage of resistant cultivars (Kinloch et al. 1985).

Much progress has been achieved in screening and breeding for resistance to root-knot nematodes, such as *M. javanica* (Treub) Chitwood, in cucumber and other *Cucumis* species, however, no progress in RKN resistance has been made (Devran et al. 2011; Fassuliotis 1970; Walters et al. 1993; Wehner et al. 1990; Winstead and Sasser 1956). Though several wild species with high resistance to RKN were identified, due to serious reproductive barriers, the attempts to transfer elite resistance to the cultivars through viable interspecific hybrids between cucumber and several related resistant wild *Cucumis* spp. have failed (Fassuliotis 1970; Nugent and Dukes 1997; Walters et al. 1993).

*Cucumis hystrix* (2n = 24) is an important wild *Cucumis* species containing valuable traits, such as resistance to RKN, downy mildew, gummy stem blight and fusarium wilts (Chen et al. 2004b), as well as tolerance to low light and temperature (Qian et al. 2002; Zhuang et al. 2002). Introgression of the elite traits from one species into another through interspecific hybridization plays an important role in cucumber genetic modification (Anamthawatjónsson 2001; Gur and Zamir 2004; Lou et al. 2013; Muir and Moyle 2009; Pang et al. 2013). IL10-1 which was identified as resistant to RKN is one of the introgression lines derived from the successful fertile interspecific hybridization between the synthesized allotetraploid species *Cucumis* × *hytivus* J.F.Chen & J.H. Kirkbr. (2n = 38, hereafter *C*. × *hytivus*, which was obtained through chromosome doubling of the interspecific hybrid between *C*. *hystrix* and *C*. *sativus*) and *C*. *sativus* (Chen and Kirkbride 2000; Chen et al. 1997; Chen et al. 2003). IL10-1, harboring the same chromosome number as cucumber (2n = 14), was a breakthrough in cucumber RKN resistance breeding.

The massively parallel next-generation sequencing (NGS) technologies bring a great opportunity for enhancement via high-throughput identification and genotyping the RIL population with a low-coverage sequencing. Huang et al. (2009a, b) developed and applied a high-throughput method, a sliding-window approach instead of single-nucleotide polymorphisms (SNPs), for genotyping of a recombinant inbred line (RIL) population derived from a cross between two sequenced rice varieties. Xie et al. (2010) developed a parent-independent strategy to construct an ultrahigh-density linkage map for genotyping a RIL population of rice based on very low coverage sequencing. Xu et al. (2013) applied the parent-independent method developed in rice into soybean to identify three QTLs to RKN resistance using a 246 RIL population derived from two low coverage sequenced parents.

In this study, we successfully constructed a linkage bin-map containing 1048 bin markers using the parent-independent method for genotyping of a recombinant inbred lines (RIL) population generated from crossing the introgression line IL10-1 with a cucumber cultivar CC3. Three different regions of the genome with QTLs for resistance to RKN were identified, in which, expression of four genes with nonsynonymous SNPs were examined as possible candidate RKN resistance genes. The results from this study will facilitate breeding for root-knot nematode resistance in cucumber.

## Materials and methods

### RIL population development

An introgression line 10-1 (IL10-1) with resistance to root-knot nematode as identified from the progenies of an interspecific cross between a wild cucumber species *Cucumis hystrix* Chakr. (2n = 24, HH) and a cultivar cucumber CC3. A RILs population, originated from an IL10-1 and the susceptible control CC3 cross, was used in this study. One hundred twenty-one F_2:6_-derived RILs were grown in two different greenhouses of Nanjing Agricultural University, Pailou experimental station and Jiangpu experimental station, respectively. Total genomic DNA was prepared from isolated young leaf tissues of RILs using the modified cetyltrimethyl ammonium bromide (CTAB) method (Murray and Thompson, 1980). The purified DNA of RILs and the two parents were then sent to SHANGHAI BIOZERON BIOTECH. CO., Ltd to construct sequencing libraries and Illumina sequencing.

### Phenotyping cucumber plants from F_2:3_ and F_2:6_ populations

**Fig. 1:**
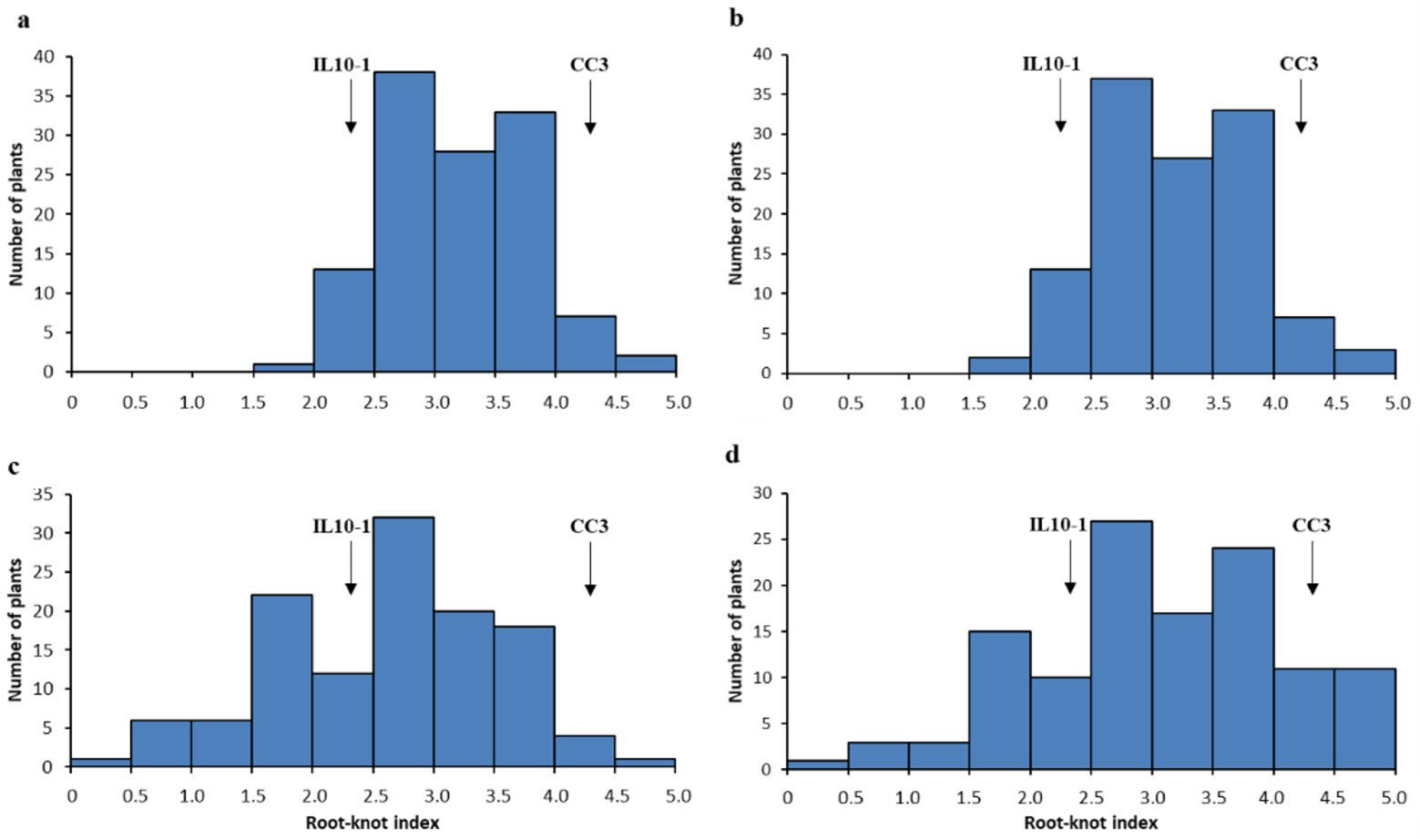
Gall index distribution of the populations derived from IL10-1 × CC3 in the different greenhouse at different seasons. **a**, **b** show gall index distribution of F2:3 in the Pailou greenhouse in 2011 spring and in the Jiangpu field in 2012 spring, respectively. **c** and d show gall index distribution of the F2:6 population in the Jiangpu greenhouse in 2014 Autumn and in the Baima greenhouse in 2015 spring, respectively. The gall index of each code was obtained by averaging the gall index of 5 plants from each family in RIL, which ranging from 0 to 5. Gall index = 0, represents immune, 0 < gall index < 3 represents resistant, gall index = 3 represents medium resistant, and 3 < gall index < 5 represents susceptible, gall index = 5 represents highly resistant. The arrows point to 2.3 and 4.3 represent the gall index of IL10-1 and CC3, respectively

The 121 individuals from F_2:3_ and F_2:6_ RILs were evaluated for RKN resistance in three greenhouses (Jiangpu, Pailou and Baima) of Nanjing Agricultural University for two seasons (Spring and Autumn), respectively (Fig. 1a, b for F_2:3_ population, while Fig. 1c, d for F_2:6_ RILs, all data are listed in Table S2). A randomized complete block experimental design with five replications was used in this study. The two-week-old seedlings were inoculated with an egg suspension at about 2000 *M. incognita* eggs per plant as described (Hussey and Barker 1973; Ibrahim et al. 2011). Plants were rated for resistance 45 d after inoculation using the gall index system (0–100% of roots galled) of Baker and Townshend (1986), and then classified into 10 grades from 0 to 5. The gall numbers in each plant were counted after washing free of soil, and gall index was calculated as described (Taylor and Sasser 1978).

### Construction of sequencing libraries and illumina sequencing

About one microgram of genomic DNA for each RIL individual was fragmented randomly into approximate 400-bp by Covaris M220. Fragmented DNA was end repaired, tailed with ‘A’ nucleotides at the 3′ end, and ligated to customized pooling barcodes and Illumina paired-end sequencing adapters. DNA fragments that had adapters on both ends were selectively enriched and amplified by PCR. The index was introduced into the adapter at this stage using primers containing index tags as appropriate.

The cucumber genome *Cucumis sativus* var. ‘Chinese long’ (http://cucurbitgenomics.org/organism/2) was used as the reference genome for this project. The genome size of ‘Chinese long’ is 196.48 Mb while the effective size is 192.92 Mb (not including the N base in the reference). The sequence depth of the 121 RILs and two parental lines was approximately 0.2 × . Additional sequences were generated from two parents to achieve > 10 × sequence depth. The qualified library was then subjected to whole-genome sequencing, resulting in over the required sequence depth for each sample. Raw image files were processed by Illumina pipeline (HiSeq 2500) for base calling with default parameters and the sequences of each individual were generated as 125-bp paired-end reads.

### SNP calling and genotyping

The cucumber genome sequence was downloaded from the Institute of Vegetables and Flowers, Chinese Academy of Agricultural Sciences (version 2.0, http://cucurbitgenomics.org/organism/2). The bioinformatics analysis begins with the sequencing data (raw data) which generated from the HiSeq. First, the raw reads with adapter sequences or too many unreliable bases (> 10 bp) will be discarded to get the useful reads (clean data). Second, the ‘clean’ reads will be mapped to the reference genome using bwa mem (http://bio-bwa.sourceforge.net/, Li and Durbin 2009). The sequencing depth and coverage compared to the reference genome were calculated based on the alignments. We used the samtools mpileup function (http://samtools.sourceforge.net) to call SNP. The population SNP information was merged, and then the merged genotype data was used to construct the genetic map using the MPR2 R package (http://crep.ncpgr.cn/supplements/MPR_genotyping/). Visualized MPR data was analyzed using the R qtl package (http://rqtl.org/).

### QTL mapping

The switch from one genotype to another within a chromosome represents a recombination event, and the transition region was defined as recombination interval. To conduct genetic analysis, we aligned the 121 RILs and defined the genomic region between any two adjacent recombination intervals within a chromosome as a bin with terminal bins exceptions, which were between a recombination interval and a chromosome end. We used bins as molecular markers to construct a linkage map using the program R/QTL package, and QTL were mapped using the MQM method of the program MapQTL 5.0 (Huang et al. 2009a, b).

### qPCR analysis of candidate genes

Root samples of IL10-1 and CC3 at early infection stage, i.e. 0, 1, 2, 3 days after inoculation of *M*. *incognita* invasion were collected for RNA analysis. The root tips from 5 plants were mixed into a single biological sample. Three biological replicates were performed. Then, the 24 total samples were immediately frozen in liquid nitrogen and stored at − 80 °C. Primer efficiency was determined using different dilution ratio (diluted 10:1) using cDNA of IL10-1, and the PCR efficiencies were about 100% (Fig. S4). Extraction of total RNAs were conducted using the TRIzol reagent (Invitrogen, Carlsbad, CA, USA). RNA was isolated from the root samples of IL10-1 and CC3 at different time-points after inoculation of RKN, and digested with DNase I (Fermentas) for 30 min at 25 °C to remove DNA according to the manufacturer’s instructions. cDNA was synthesized from 5 mg of total RNA by using a cDNA Synthesis Kit (Fermentas). RT-qPCR was carried out using the LightCycler 480 Real-Time PCR System (Roche, Germany). The Ct value of each gene was investigated and normalized to the Ct value of *EF1a*, which is our reference gene (Wan et al. 2010). The formula 2^−ΔΔCt^ was applied to determine relative expression fold differences for each gene after inoculation, and for the specific algorithm see the details in Li et al. (2014). Specific primers for each candidate gene were designed using Primer 3 program (Untergasser et al. 2007). PCR products amplified from these primers were sequenced to determine if the correct genes of interest were amplified. Only primer pairs that give a single amplicon were selected for RT-qPCR (Table S1). The RT-qPCR reactions were conducted with three technical replications in 10 μl reaction for each biological replicate using Qiagen QuantiFast SYBR Green RT-PCR Kit (Qiagen, Valencia CA). Following the reverse transcriptase reaction, amplification was conducted at 95 °C for 15 min, then 35 cycles of 95 °C for 20 s, 60 °C for 20 s, and 72 °C for 20 s.

## Results

### Phenotyping the population plants to RKN (*M*. *incognita* Chitwood) Resistance

**Fig. 2:**
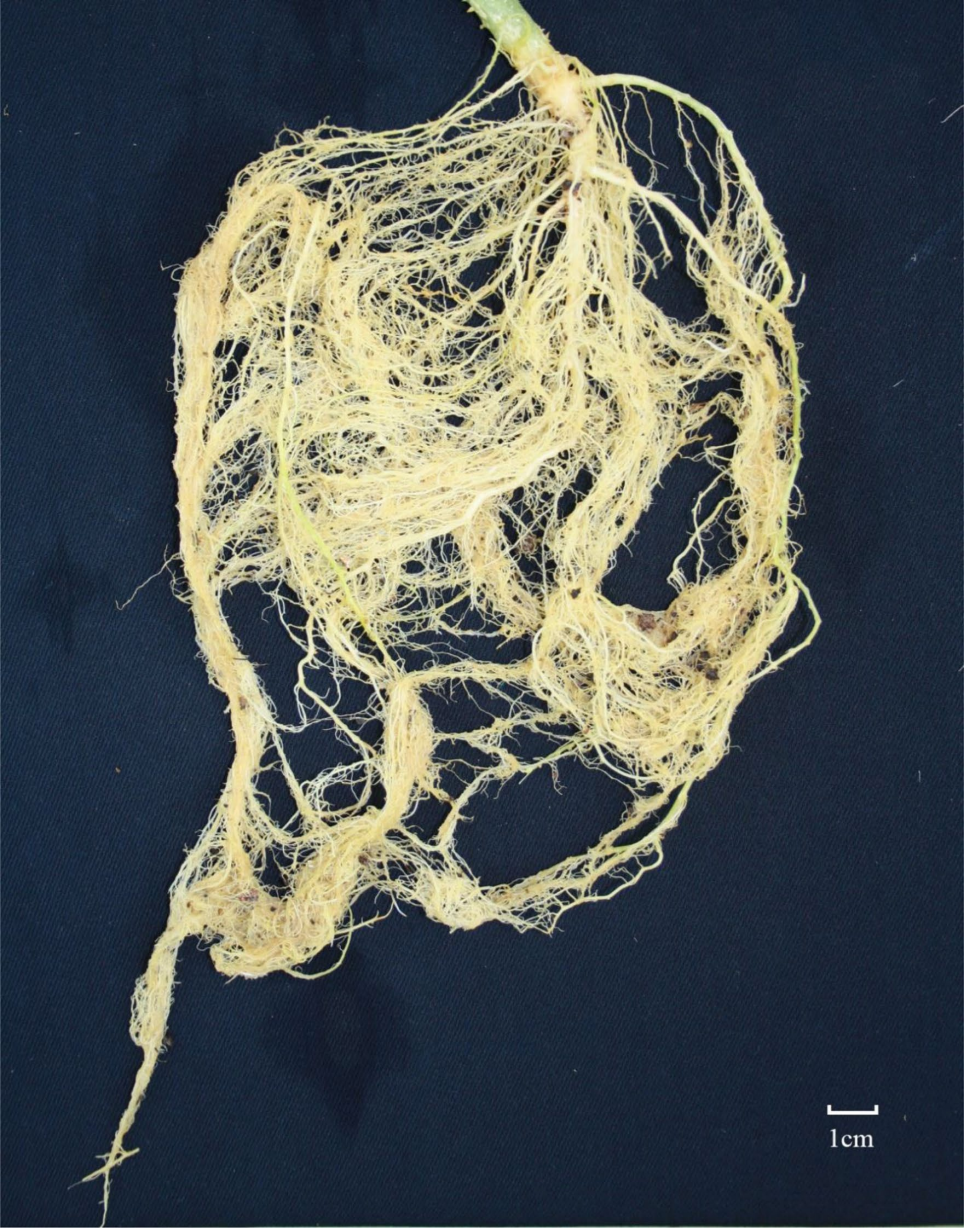
Root symptoms of C. *hystrix* Chakr. after 45 days inoculation with *Meloidogyne incognita*. Bar = 1.0 cm

Previously twenty-one introgression lines (ILs), together with one cucumber cultivar and one wild cucumber species *C. hystrix* (Fig. 2, showing the root phenotyping of *C. hystrix*), were tested for RKN resistance in the summer of 2009 in the greenhouse at Pailou experimental base, Nanjing Agricultural University (Table 1). All the ILs were selfed for generations to obtain relatively stable pure lines. According to a modified version of the gall index system used by Taylor and Sasser (1978), the ILs and cultivars were harvested after 45 days of inoculation and were scored for mean number of galls and disease level. Introgression line IL10-1, derived from HH_z_1-12-24, and cultivar CC3 were used as the resistant and susceptible controls, respectively (Fig. S1) (Cheng 2016). And a continuous distribution from resistance to susceptible was obtained by calculating the gall index of the F_2:3_ population, which was the predominant indicator for resistance to RKN, suggesting polygenic control of RKN-resistance (Fig. 1a, b, Table S2). The gall index of the RIL population was also recorded by rating root galling in two greenhouses at two different planting seasons (Fig. 1c, d, Table S2) which was later used in the QTL mapping work.

**Table 1:** Resistance identification of cucumber introgression lines to *M. incognita*

Code	Cultivars or lines	Root-knot index	Resistant classification	Code	Cultivars or lines	Root-knot index	Resistant classification
1	*Cucumis hystrix*	1.35 ± 0.11	R	13	HH1-17	4.04 ± 0.41	HS
2	CC3	4.84 ± 0.16	HS	14	HH1-18	3.25 ± 0.13	S
3	HH1-2*	2.78 ± 0.11	MR	15	HH1-19	4.68 ± 0.14	S
4	HH1-6	4.28 ± 0.43	HS	16	HH_Z_1-12-24**	2.86 ± 0.16	MR
5	HH1-7	4.52 ± 0.03	HS	17	HH_Z_1-14-35-10	3.75 ± 0.14	S
6	HH1-8	4.54 ± 0.42	HS	18	HH_Z_1-14-38	4.0 ± 0.09	HS
7	HH1-9	4.86 ± 0.01	HS	19	HH_Z_1-13-29	4.38 ± 0.08	HS
8	HH1-11	2.86 ± 0.01	MR	20	HH_Z_1-16-13-28	3.63 ± 0.17	S
9	HH1-12	4.84 ± 0.17	HS	21	HH_Z_1-1-30	4.33 ± 0.14	HS
10	HH1-13	4.38 ± 0.38	HS	22	HH_Z_1-6-10	4.42 ± 0.44	HS
11	HH1-14	4.54 ± 0.35	HS	23	HH_Z_1-1-9	4.77 ± 0.21	HS
12	HH1-16	3.63 ± 0.17	S	24	HH_Z_3-18-9	4.75 ± 0.32	HS

*R* resistant, *MR* medium Resistant, *S* susceptible, *HS* highly susceptible. *Cucumis hystrix* was used as the resistant control, CC3 as the susceptible control

*, **The two importance medium resistant introgression line derived from hybridization between Cucumis hystrix and CC3

**The original material selfing to harvest IL10-1

### Construction of bin and genetic linkage maps of recombinant inbred lines

A high throughput genotyping approach based on Illumina sequencing was used to construct the bin-map of the recombinant inbred lines. 121 RILs derived from a cross between IL10-1 and CC3 were used to construct the DNA libraries. A total of 486,807,778 reads were generated with an average of 3,990,228 reads per RIL individual (SRP211885). The effective sequence depths for each RIL ranged from 0.78-fold to 2.02-fold coverage of the cucumber genome with an average of 1.20-fold coverage. The corresponding sequence coverage ranged from 34.06 to 68.64% with a mean of 16.2% for each RIL individual. The reads of both parents were aligned with the Cucumber ‘Chinese long’ genome sequence (Version 2) (Huang et al. 2009a, b). At the same time, the physical positions of 510,456 SNPs were identified from CC3 and 532,639 SNPs from IL10-1.

**Fig. 3:**
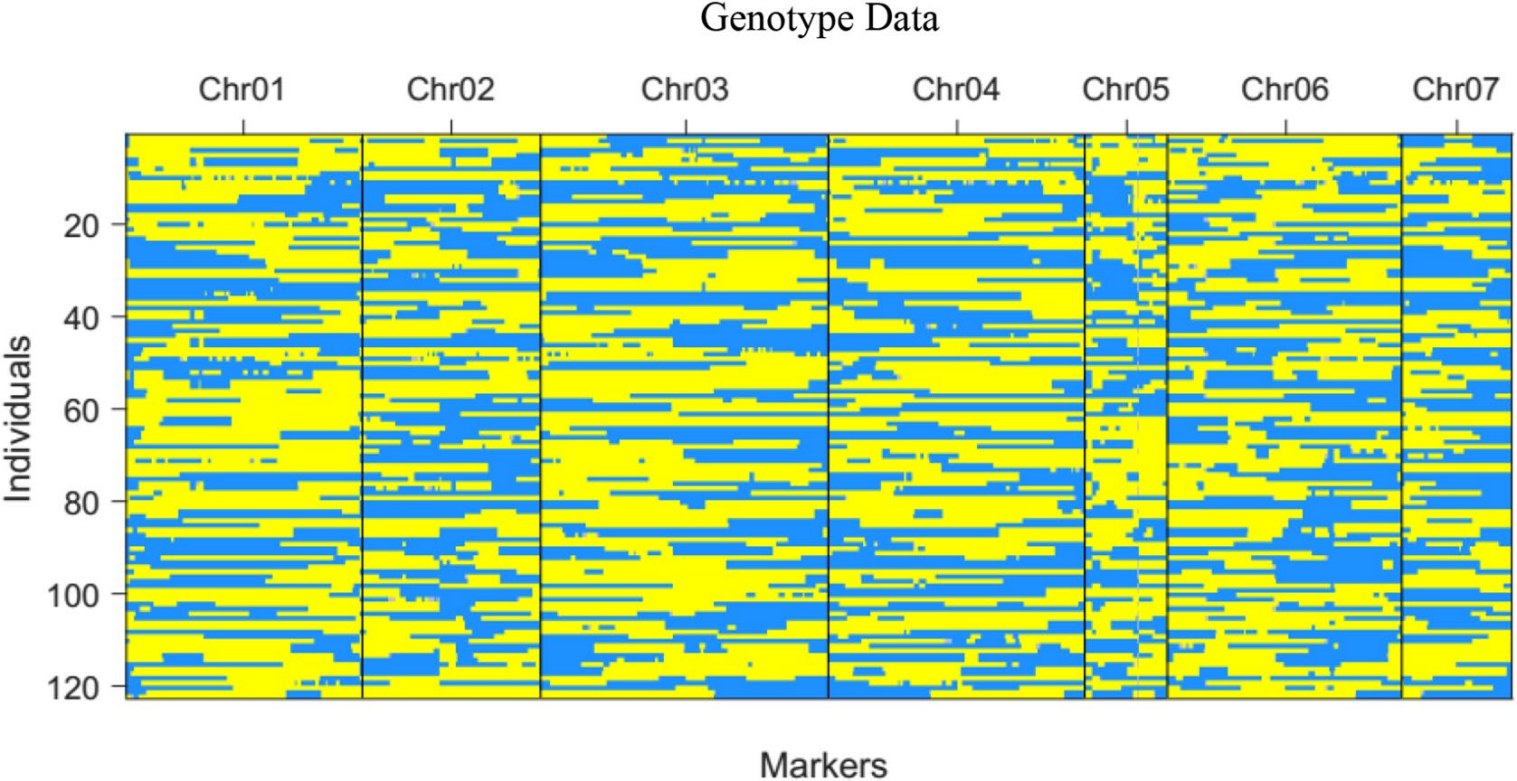
Recombination bin-map of RIL population. Recombination bin-map of RIL population. Bin-map consists of 1048 bin markers inferring from 248,168 high quality SNPs in RIL population. Physical position is based on ‘Chinese Long’ genome sequence (Version2). Blue: IL10-1 genotype; Yellow: CC3 genotype

Based on the individual SNPs, bin map was constructed for all 121 RILs, and the chromosome fragment between two adjacent recombination intervals was defined as a bin, resulting in a skeleton bin map consisting of 1048 recombinant bins distributed throughout the genome (Table 2, Fig. 3). The estimated recombination fraction and LOD scores for all pairs of markers were calculated to diagnose the quality of genetic map construction (Fig. S2). The number of bins on each chromosome ranged from 90 to 217, and the length of average interval between two bin markers on each chromosome ranged from 19.13 to 39.77 Mb, with a mean interval of 1.94 Mb. Regarding each bin as a marker, the genetic linkage map of the RIL population was constructed based on the recombination frequency. The total length of the linkage map for the RIL population was 1070.71 cM, while the genetic lengths of the linkage groups ranged from 80.59 to 277.35 cM (Table 2, Fig. S3).

**Table 2:** Distribution of bin-markers among seven chromosomes established on a genetic linkage bin-map using an RIL population derived from the cross IL10-1 × CC3

Chromosome code	Number of bin markers	Average interval (kb)	Chromosome size (kp)
1	157	184.863	29023.435
2	146	158.617	23158.145
3	217	183.289	39773.774
4	190	123.267	23420.722
5	90	311.191	28007.172
6	156	186.187	29045.234
7	92	207.923	19128.873

### High-density-marker QTL mapping of cucumber for RKN resistant trait

**Fig. 4:**
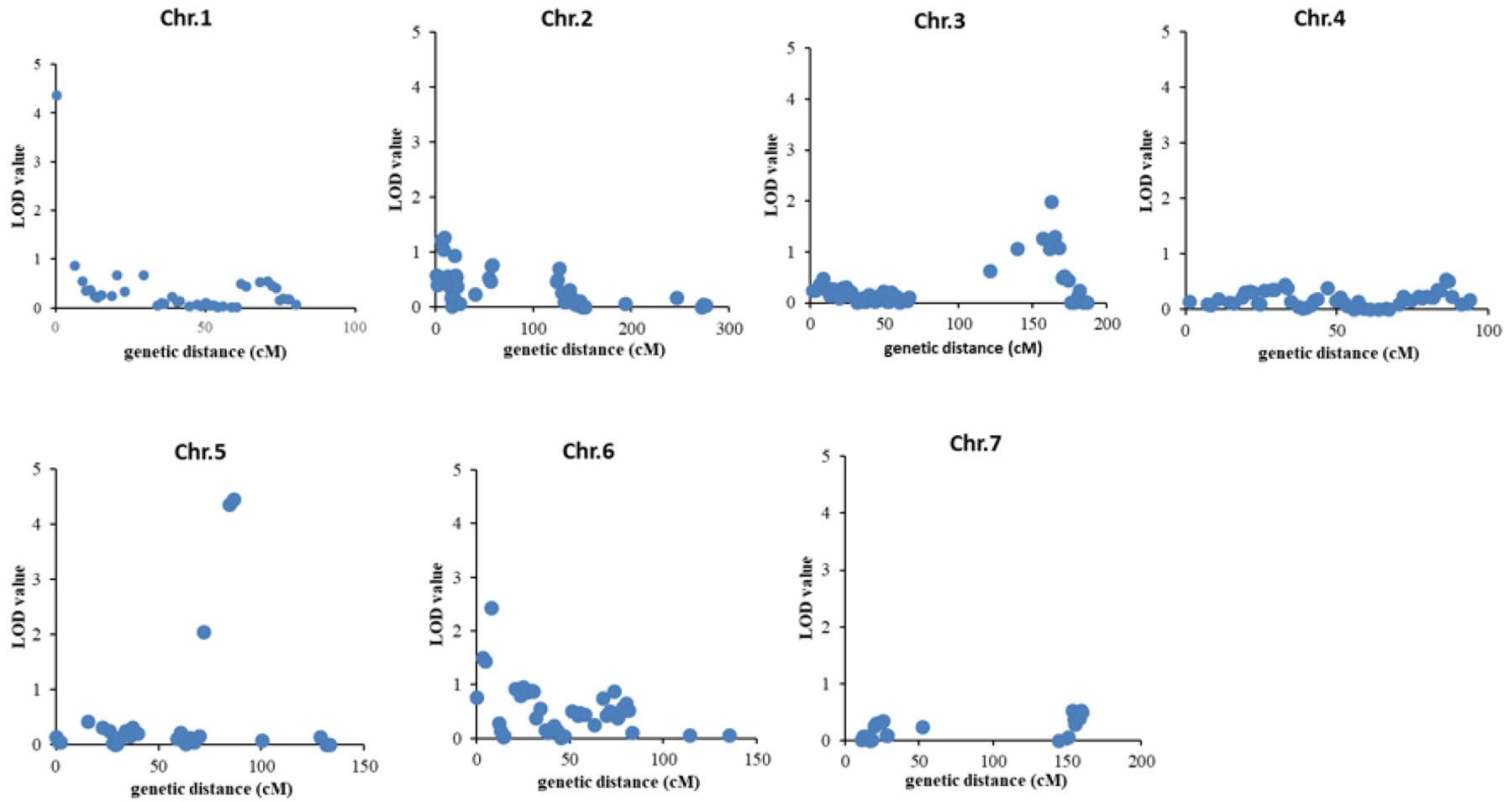
Genetic map of QTLs on cucumber chromosomes. Genomic region between any two adjacent recombination intervals within a chromosome were defined as a bin. Bins were used as molecular markers to construct a linkage map using the program R/QTL package, and QTLs were mapped using the MQM method of the program MapQTL 5.0

Both the gall index distribution data of the RIL populations from two different greenhouses at two different planting seasons (Baima greenhouse in 2015 spring and Jiangpu greenhouse in 2014 Autumn) showed a continuous distribution from resistant to susceptible, indicating polygenic control of RKN-resistance (Fig. 1c, d, Table S2). Three QTLs for RKN resistance were identified in these two replicate experiments by multidimensional quality metric (MQM) (Wang et al. 2007). One QTL (qRKN1-1) was mapped to bin marker C01B2 of chromosome 1 with a logarithm of odds (LOD) score of 4.36 (0.41 cM), explaining 13.36% of total genetic variance (Figs. S3, 4). The additive effect of this QTL was 0.29. The other two QTLs (qRKN5-1 and qRKN5-2) were mapped to C05B45 and C05B46 of chromosome 5 with an LOD score of 4.35 (84.24 cM) and 4.44 (84.65 cM) respectively, which explained 9.07% and 9.58% of the total phenotypic variance respectively (Fig. 4, Table 3).

**Table 3:** Biometrical parameters of identified QTLs based on whole-genome resequencing approach

Chromosome location	bin marker	Position (Mb)	bin marker	LOD	R2 (%)	Bin size (kb)
Chr.1	C01B2	0.41	C01B2	4.3591	13.36	7
Chr.5	C05B45	84.24	C05B45	4.3536	9.07	176
Chr.5	C05B46	84.65	C05B46	4.4422	9.58	175

### Identifying of the possible causal genes underlying the QTLs for RKN resistance

Through the comparative analysis of the genomes of IL10-1, CC3 and the RIL individuals, a sliding-window approach was applied to search for candidate genes for RKN in the QTL intervals. Comparing sequencing of IL10-1 and CC3, we found 67 genes in total with single nucleotide variation (SNV) in the bin marker section for three QTL, including 37 genes with nonsynonymous SNPs from chromosome 1 and chromosome 5 in IL10-1 (Table 4). Combining the current annotation information of *Cucumis* cucumber line 9930 (http://cucurbitgenomics.org/) and *Arabidopsis TAIR* (http://www.arabidopsis.org/), using software Annovar (Wang et al. 2010), annotation was assigned for the majority of the genes.

**Table 4:** List of 37 candidate genes for the main-effect QTLs based on RIL re-sequencing

Chromosome	Mutation	Gene ID	Ortholog	Annotation
Chr.1	C→G	*Csa1M004180.1*	–	WD repeat-containing protein 65-like
Chr5	G→A	*Csa5M606820.1*	*AT2G26710.1*	BAS1 (PHYB ACTIVATION TAGGED SUPPRESSOR 1)
Chr5	A→T	*Csa5M606830.1*	*AT2G26710.1*	BAS1 (PHYB ACTIVATION TAGGED SUPPRESSOR 1)
Chr5	G→A	*Csa5M606840.1*	*AT2G26710.1*	BAS1 (PHYB ACTIVATION TAGGED SUPPRESSOR 1)
Chr5	G→A	*Csa5M606850.1*	*AT2G03810.1*	18S pre-ribosomal assembly protein gar2-related
Chr5	A→G	*Csa5M606880.1*	*AT1G69020.1*	Prolyl oligopeptidase family protein
Chr5	A→T	*Csa5M606910.1*	*AT2G03890.1*	Hosphatidylinositol 3- and 4-kinase family protein
Chr5	A→T	*Csa5M606920.1*	*AT1G13635.1*	Methyladenine glycosylase family protein
Chr5	C→A	*Csa5M607420.1*	*AT2G24120.1*	SCA3 (SCABRA 3)
Chr5	C→G	*Csa5M607450.1*	–	–
Chr5	G→A	*Csa5M607990.1*	*AT1G26230.1*	Chaperonin
Chr5	C→G	*Csa5M608220.1*	*AT3G25640.1*	Unknown protein
Chr5	A→T	*Csa5M608240.1*	*AT3G25670.1*	Leucine-rich repeat family protein
Chr5	T→C	*Csa5M608250.1*	*AT1G26110.2*	Unknown protein
Chr5	T→C	*Csa5M608260.1*	*AT1G26100.1*	Cytochrome B561 family protein
Chr5	A→C	*Csa5M608270.1*	*AT3G25680.1*	Unknown protein
Chr5	C→A	*Csa5M608280.2*	*AT3G25690.1*	CHUP1 (CHLOROPLAST UNUSUAL POSITIONING 1)
Chr5	T→C	*Csa5M608290.1*	*AT1G26090.1*	Unknown protein
Chr5	T→C	*Csa5M608320.1*	*AT1G68800.1*	TCP12 (TCP DOMAIN PROTEIN 12)
Chr5	A→T	*Csa5M608330.1*	*AT1G68810.1*	bHLH family protein
Chr5	C→G	*Csa5M608340.1*	*AT5G08550.1*	ILP1 (increased level of polyploidy1-1D)
Chr5	A→T	*Csa5M608360.1*	*AT1G13245.1*	RTFL17 (ROTUNDIFOLIA LIKE 17)
Chr5	T→A	*Csa5M610370.1*	*AT5G38280.1*	PR5 K
Chr5	T→C	*Csa5M610420.1*	*AT2G01210.1*	Leucine-rich repeat transmembrane protein kinase
Chr5	A→T	*Csa5M612260.1*	–	–
Chr5	A→T	*Csa5M612850.1*	*AT2G35840.1*	Sucrose-phosphatase 1 (SPP1)
Chr5	A→G	*Csa5M612860.1*	*AT5G16010.1*	3-oxo-5-alpha-steroid 4-dehydrogenase family protein
Chr5	A→C	*Csa5M613560.1*	*AT2G36050.1*	OFP15 (ARABIDOPSIS THALIANA OVATE FAMILY PROTEIN 15)
Chr5	G→A	*Csa5M615180.1*	*AT5G05100.1*	Nucleic acid binding
Chr5	G→A	*Csa5M616330.1*	*AT1G08230.2*	Amino acid transporter family protein
Chr5	G→C	*Csa5M622630.1*	*AT1G75950.1*	SKP1 (S PHASE KINASE-ASSOCIATED PROTEIN 1)
Chr5	A→T	*Csa5M622680.1*	*AT3G10360.1*	APUM4 (Arabidopsis Pumilio 4)
Chr5	G→A	*Csa5M622780.1*	*AT5G04220.2*	SYTC
Chr5	C→G	*Csa5M622850.1*	–	–
Chr5	A→G	*Csa5M622860.2*	*AT5G13570.1*	DCP2 (DECAPPING 2)
Chr5	G→A	*Csa5M623410.1*	*AT4G23170.1*	Programmed cell death, response to salicylic acid, systemic acquired resistance
Chr5	G→A	*Csa5M623490.1*	*AT3G07990.1*	SCPL27 (serine carboxypeptidase-like 27)

**Fig. 5:**
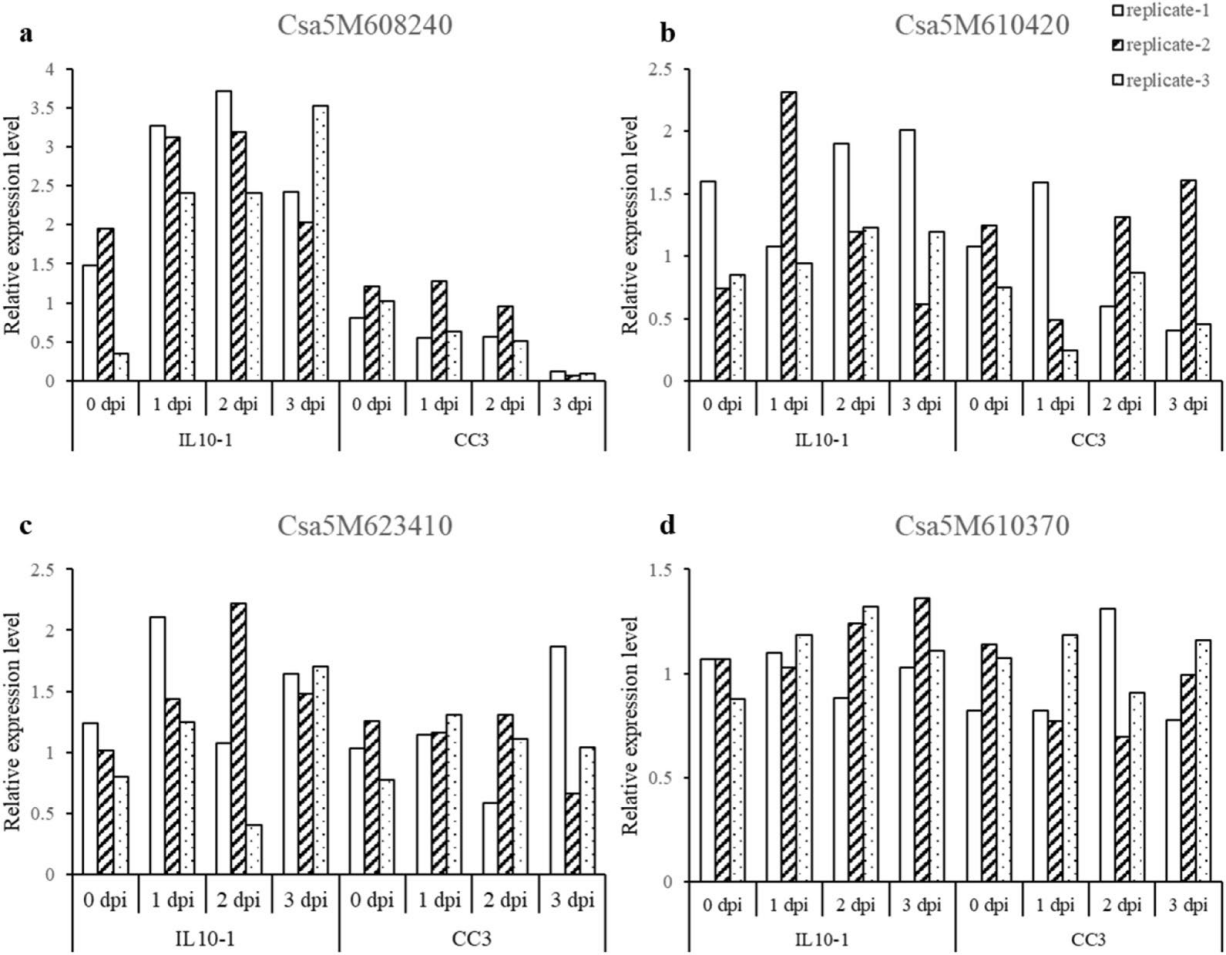
Expression pattern of four probable genes in IL10-1 and CC3 at early infection stage of nematode invasion

According to the annotations, among the 37 changes influencing coding regions, four of them, *Csa5M608240.1*, *Csa5M610420.1*, *Csa5M623410.1* and *Csa5M610370.1* from chromosome 5, were genes that have been associated with disease resistance in other systems likely to be the candidate RKN-resistant related genes (Table S3). Gene *Csa5M608240.1* and *Csa5M610420.1* are predicted to encode a leucine-rich repeat family protein and a leucine-rich repeat transmembrane protein kinase, respectively. Gene *Csa5M623410.1* is predicted to encode the proteins involving in programmed cell death, response to salicylic acid, systemic acquired resistance. And gene *Csa5M610370.1* is with annotation of pathogenesis related 5-like receptor kinase. Gene-expression of root tissues studies revealed that these four genes showed different expressing models at the early-inoculated stage in IL10-1 and CC3 (Fig. 5). Gene *Csa5M608240.1* was induced significantly in IL10-1 at the first 3 days after RKN inoculation, while light inhibition was observed in CC3 during the first 2 days after RKN inoculation and nothing was observed at the third day post inoculation (Fig. 5a). Gene *Csa5M610420.1* was also induced significantly in IL10-1 but was suppressed in CC3 at the early-inoculated stage (Fig. 5b). However, gene *Csa5M623410.1* and gene *Csa5M610370.1* showed the same expressing trend in the two materials and had less significant differences between IL10-1 and CC3, which need to be confirmed in further function identification by transformation systems (Fig. 5c, d).

## Discussion

RKN, an economically important plant root parasite, is the most common and destructive nematode for cucumber and other crops, including cotton, soybean, and peanut. The most environmentally friendly and cost-effective strategy to control RKN is to develop cucumber cultivars with multiple and durable RKN resistance. However, there is no RKN resistant cucumber cultivar currently available. Wild genetic resources, harboring elite resistant genes, play an important role in the crop improvement and the development of agriculture (Hammer 2004). Exploiting those resistances from wild germplasm is the most economical approach to reduce yield loss caused by RKN in cucumber and most other crops. *Cucumis hystrix* Chakr. (2n = 24, HH) is an important wild species in *Cucumis* containing valuable traits for cucumber improvement. In order to introgress those elite traits of *C*. *hystrix* into *C*. *sativus*, introgression lines (ILs) have been verified to be useful tools for the QTLs mapping, and individual QTL effect study (Gur and Zamir 2004; Muir and Moyle 2009; Tian et al. 2006).

Excavating candidate resistant gene (s) of the ILs is conducive to uncover knowledge about the mechanism of the resistance (Huang et al. 2013; Qiu et al. 2012; Tian et al. 2006). Also, it is the basis for the development of functional diagnostic markers for effective marker assisted selection to incorporate the trait into elite cucumber cultivars. In our previous study, ILs with resistance to different pathogens were generated and assayed after the interspecific hybridization between *C*. *hystrix* and *C*. *sativus* var ‘Beijingjietou’ CC3 (Chen et al. 2004a). Since cultivated cucumber possesses low genetic diversity, these ILs derived from wild cucumber *C*. *hystrix* are a useful bridge to provide a broader genetic basis for cucumber improvement (Dijkhuizen et al. 1996; Pląder et al. 2007).

A RIL population, derived from a cross between the resistant introgression line IL10-1 and the susceptible cultivar CC3, was used to map RKN-resistance QTLs. We have successfully introduced the method developed for the RKN resistance gene mapping in rice to construct an ultrahigh-density genetic linkage map of high-quality SNPs based on very low depth (0.05×) sequences of the cucumber RILs (Xie et al. 2010). In this study, a skeleton bin map consisting of 1048 recombinant bins in the F_2:6_ population (Fig. 1c, d). The phenotypic distribution of root-knot index in two different greenhouses both were an approximately normal distribution but with different tracer curve types, which indicated not only that a few genes with potentially large effects may be present in the IL10-1, but also that environmental effects play a role in the effect of RKN resistance. Thus, more and larger population needs to be constructed to further confirm the effect of the identified QTLs.

Although the whole genome sequence of cucumber was released in 2009, and hundreds of SSR markers have been developed, less than 13% of the SSR with polymorphism between the two parents could be applied in our population (data not shown). Currently, SNPs have been widely developed and applied in genotyping through next-generation sequencing (NGS) technologies. Different from traditional SNP discovery approaches, we applied an advanced approach developed in the mapping research in rice (Xie et al. 2010), wherein both parental lines were re-sequenced at a higher coverage, while the individuals RILs were sequenced at very low depth (0.05×).

A set of 27,705 phased SNPs identified in parental lines were selected to genotype the RIL population under stringent criteria. The bin map was constructed based on these SNPs. The concordance test was conducted between the genetic map and the genome sequence data of cucumber assembly 2.0, as well as with the sequenced data of the wild species *C*. *hystrix* (Qin et al. unpublished) to further validate the SNPs. As expected, the mapping resolution in pericentromeric regions was not significantly different than that on chromosome arms, while resolution at the chromosome ends was relatively low due to the large bin size resulting from recombination suppression and high gene density (Fig. S3) (Xu et al. 2013). But there are still exceptions in our research such as the low resolution of bin markers distributed on Chromosome 5 (Fig. S3). So, to precisely map genes/QTL in the terminal regions of the chromosomes, a larger RIL population needs to be constructed.

Three QTL regions, one located on chromosome 1 and two located on chromosome 5 of cucumber introgression line IL10-1 were identified based on ultrahigh-density genetic linkage maps of high-quality SNPs. Thirty-seven genes with nonsynonymous SNPs were identified in these three QTL regions. Of those, four genes were considered as candidate genes for RKN resistance. In which, gene *Csa5M608240* and *Csa5M610420*, encoding a leucine-rich repeat family protein, and a leucine-rich repeat receptor protein kinase-like protein, respectively. Leucine-rich repeat family protein and leucine-rich repeat receptor protein kinase-like protein both contain the same structural motif. Leucine-rich repeat (LRR), which is characterized to be a structural motif used in molecular recognition processes (Bent et al. 1994; Kobe and Deisenhofer 1995), and is a structural component of the largest R-gene family among plant genomes (Saintenac et al. 2018; Wan et al. 2013; Yuan et al. 2018; Zhang et al. 2011). Interesting, we found there are two genes *Csa5M623410* and *Csa5M610370* annotated as encoding PCD protein (programmed cell death protein, response to salicylic acid, systemic acquired resistance) and the PR5 K (pathogenesis related 5-like receptor kinase) which did not show any difference between our resistant IL10-1 and susceptible CC3 after inoculation of RKN (Fig. 5d). The PCD protein and the PR5 K should be involved in disease resistance (Greenberg and Yao 2004; Wang et al. 1996), and be a candidate gene performing difference. However, it is currently unknown whether these four genes in our study contribute to cucumber RKN resistance. Cloning and characterization of the candidate genes will ultimately be needed to test their functionality in resistance to RKN.

## Electronic supplementary material

Below is the link to the electronic supplementary material.

Supplementary material 1 (PDF 1563 kb)

## References

[CR1] Anamthawatjónsson K (2001). Molecular cytogenetics of introgressive hybridization in plants. Method Cell Sci.

[CR2] Baker KR, Townshend JL, et al (1986) Determining nematode population responses to control agents. Methods for Evaluating Pesticides for Control of Plant Pathogenes, pp 283–296

[CR3] Bent AF, Kunkel BN, Dahlbeck D (1994). RPS2 of *Arabidopsis thaliana*: a leucine-rich repeat class of plant disease resistance genes. Science.

[CR5] Chen J, Kirkbride J (2000). A new synthetic species of *Cucumis* (Cucurbitaceae) from interspecific hybridization and chromosome doubling. Brittonia.

[CR6] Chen J, Staub J, Tashiro Y (1997). Successful interspecific hybridization between *Cucumis sativus* L. and *C. hystrix* Chakr. Euphytica.

[CR7] Chen J, Staub J, Qian C (2003). Reproduction and cytogenetic characterization of interspecific hybrids derived from *Cucumis hystrix* Chakr. × *Cucumis sativus* L. Theor Appl Genet.

[CR8] Chen J, Luo X, Qian C (2004). *Cucumis* monosomic alien addition lines: morphological, cytological, and genotypic analyses. Theor Appl Genet.

[CR9] Chen J, Moriarty G, Jahn M et al (2004b) Some disease resistance tests in *Cucumis hystrix* and its progenies from interspecific hybridization with cucumber. In: Meeting on progress in *Cucurbit* genetics and breeding research Proceedings of Cucurbitaceae

[CR10] Cheng C (2016) Studies on genetics and mechanism of the resistance to southern root-knot nematode in *Cucumis sativus*-*hystrix* introgression line. Dissertation, Nanjing Agricultural University

[CR11] Devran Z, Firat A, Tör M (2011). AFLP and SRAP markers linked to the mj gene for root-knot nematode resistance in cucumber. Sci Agric.

[CR12] Dijkhuizen A, Kennard W, Havey M (1996). RFLP variation and genetic relationships in cultivated cucumber. Euphytica.

[CR13] Fassuliotis G (1970). Resistance of *Cucumis* spp. to the Root-knot Nematode, *Meloidogyne incognita* acrita. J Nematol.

[CR14] Greenberg T, Yao N (2004). The role and regulation of programmed cell death in plant–pathogen interactions. Cell Microbiol.

[CR15] Gur A, Zamir D (2004). Unused natural variation can lift yield barriers in plant breeding. PLoS Biol.

[CR02] Hammer K (2004). Resolving the challenge posed by agrobiodiversity and plant genetic resources—an attempt. J Agr Rural Dev Trop Subtrop Beiheft.

[CR16] Huang S, Li R, Zhang Z (2009). The genome of the cucumber, *Cucumis sativus* L. Nat Genet.

[CR17] Huang X, Feng Q, Qian Q (2009). High-throughput genotyping by whole-genome resequencing. Genome Res.

[CR18] Huang D, Qiu Y, Zhang Y (2013). Fine mapping and characterization of BPH27, a brown planthopper resistance gene from wild rice (*Oryza rufipogon* Griff.). Theor Appl Genet.

[CR19] Hussey R, Barker K (1973). A comparison of methods of collecting inocula of *Meloidogyne* spp., including a new technique. Plant dis rep.

[CR20] Ibrahim H, Hosseini P, Alkharouf N (2011). Analysis of Gene expression in soybean (*Glycine max*) roots in response to the root knot nematode *Meloidogyne incognita* using microarrays and KEGG pathways. BMC Genomics.

[CR21] Kinloch RA, Hiebsch CK, Peacock HA (1985). Comparative root-knot galling and yield responses of soybean cultivars to *Meloidogyne incognita*. Plant Dis.

[CR22] Kobe B, Deisenhofer J (1995). A structural basis of the interactions between leucine-rich repeats and protein ligands. Nature.

[CR01] Li H, Durbin R (2009). Fast and accurate short read alignment with Burrows–Wheeler transform. Bioinformatics.

[CR24] Li J, Wu Z, Cui L (2014). Transcriptome comparison of global distinctive features between pollination and parthenocarpic fruit set reveals transcriptional phytohormone cross-talk in cucumber (*Cucumis sativus* L.). Plant Cell Physiol.

[CR25] Lou L, Wang H, Qian C (2013). Genetic mapping of gummy stem blight (*Didymella bryoniae*) resistance genes in *Cucumis sativus*-*hystrix* introgression lines. Euphytica.

[CR26] Ma J, Mao Z, Li H (2014). Resistance identification of *Cucumis metuliferus* to *Meloidogyne incognita* and characteristic analysis. Acta Hortic Sin.

[CR27] Milligan SB, Bodeau J, Yaghoobi J (1998). The root knot nematode resistance gene *Mi* from tomato is a member of the leucine zipper, nucleotide binding, leucine-rich repeat family of plant genes. Plant Cell.

[CR28] Muir C, Moyle L (2009). Antagonistic epistasis for ecophysiological trait differences between *Solanum* species. New Phytol.

[CR29] Murray HG, Thompson WF (1980). Rapid isolation of higher weight DNA. Nucleic Acids Res.

[CR30] Nugent P, Dukes P (1997). Root-knot nematode resistance in *Cucumis* species. J Agric Food Chem.

[CR31] Pang X, Zhou X, Wan H (2013). QTL Mapping of Downy Mildew Resistance in an Introgression Line Derived from Interspecific Hybridization Between Cucumber and *Cucumis hystrix*. J Phytopathol.

[CR32] Pham A, Abdel-Haleem H, Boerma H (2013). Fine mapping and identification of candidate genes controlling the resistance to southern root-knot nematode in PI 96354. Theor Appl Genet.

[CR33] Pląder W, Yukawa Y, Sugiura M (2007). The complete structure of the cucumber (*Cucumis sativus* L.) chloroplast genome: its composition and comparative analysis. Cell Mol Biol Lett.

[CR34] Qian C, Chen J, Zhuang F (2002). Several photosynthetic characters of the hybrisd species *Cucumis hytivus* under weak light condition. Plant Physiol Commun.

[CR35] Qiu Y, Guo J, Jing S (2012). Development and characterization of japonica rice lines carrying the brown planthopper-resistance genes *BPH12* and *BPH6*. Theor Appl Genet.

[CR36] Saintenac C, Lee W, Cambon F (2018). Wheat receptor-kinase-like protein Stb6 controls gene-for-gene resistance to fungal pathogen *Zymoseptoria tritici*. Nat Genet.

[CR37] Shukla N, Yadav R, Kaur P (2017). Transcriptome analysis of root-knot nematode (*Meloidogyne incognita*)-infected tomato (*Solanum lycopersicum*) roots reveals complex gene expression profiles and metabolic networks of both host and nematode during susceptible and resistance responses. Mol Plant Pathol.

[CR38] Taylor AL, Sasser JN (1978) Biology, identification and control of root-knot nematodes (*Meloidogyne* species). In: Eds: North Carolina State University Graphics, p 111

[CR39] Tian F, Li D, Zhu Z (2006). Construction of introgression lines carrying wild rice (*Oryza rufipogon* Griff.) segments in cultivated rice (*Oryza sativa* L.) background and characterization of introgressed segments associated with yield-related traits. Theor Appl Genet.

[CR40] Untergasser A, Nijveen H, Rao X (2007). Primer3Plus, an enhanced web interface to Primer3. Nucleic Acids Res.

[CR41] Walters S, Wehner T, Barker K (1993). Root-knot nematode resistance in cucumber and horned cucumber. HortSci.

[CR42] Wan H, Zhao Z, Qian C (2010). Selection of appropriate reference genes for gene expression studies by quantitative real-time polymerase chain reaction in cucumber. Anal Biochem.

[CR43] Wan H, Yuan W, Bo K (2013). Genome-wide analysis of NBS-encoding disease resistance genes in *Cucumis sativus* and phylogenetic study of NBS-encoding genes in Cucurbitaceae crops. BMC Genomics.

[CR44] Wang X, Zafian P, Lawton M (1996). The PR5 K receptor protein kinase from *Arabidopsis thaliana* is structurally related to a family of plant defense proteins. PNAS.

[CR45] Wang J, Koehler K, Dekkers J (2007). Interval mapping of multiple quantitative trait loci. Genet Sel Evol.

[CR46] Wang K, Li M, Hakonarson H (2010). ANNOVAR: functional annotation of genetic variants from next-generation sequencing data. Nucleic Acids Res.

[CR47] Wehner TC, Walters SA, Barker KR (1990) Root knot nematode egg concentration for inoculating *Cucumis* spp. tests. Report—Cucurbit Genetics Cooperative, pp 8–9

[CR48] Winstead N, Sasser J (1956). Reaction of cucumber varieties to five root-knot nematodes (*Meloidogyne* spp.). Plant Dis Rep.

[CR49] Xie W, Feng Q, Yu H (2010). Parent-independent genotyping for constructing an ultrahigh-density linkage map based on population sequencing. PNAS.

[CR50] Xu X, Zeng L, Tao Y (2013). Pinpointing genes underlying the quantitative trait loci for root-knot nematode resistance in palaeopolyploid soybean by whole genome resequencing. PNAS.

[CR51] Yuan N, Yuan S, Li Z (2018). STRESS INDUCED FACTOR 2, a leucine-rich repeat kinase regulates basal plant pathogen defense. Plant Physiol.

[CR52] Zhang H, Cao Y, Zhao J (2011). A pair of orthologs of a leucine-rich repeat receptor kinase-like disease resistance gene family regulates rice response to raised temperature. BMC Plant Biol.

[CR53] Zhuang F, Chen J, Qian C (2002). Responses of seedlings of *Cucumis *×* hytivus* and progenies to low temperature. J NAU.

