# Supplementary material for: Candidate genes underlying the quantitative trait loci for root-knot nematode resistance in a Cucumis hystrix introgression line of cucumber based on population sequencing
Source: J Plant Res. 2019 Oct 25;132(6):813–23. doi: 10.1007/s10265-019-01147-1 (PMC6831543; doi:10.1007/s10265-019-01147-1)
Supplement: Supplementary file 1 — Supplementary material 1 (PDF 1563 kb) [file 10265_2019_1147_MOESM1_ESM.pdf]

Electronic supplementary materials

Title:

Candidate genes underlying the quantitative trait loci for root-knot nematode resistance in a *Cucumis hystrix* introgression line of cucumber based on population sequencing

Authors:

Chunyan Cheng, Xing Wang, Xuejiao Liu, Shuqiong Yang, Xiaqing Yu, Chuntao Qian, Ji Li, Qunfeng Lou, Jinfeng Chen

Journal:

Journal of Plant Research

Corresponding author:

Jinfeng Chen

(College of Horticulture, Nanjing Agricultural University, No.1 Weigang Street, Nanjing, 210095, China)

Tel: +00-86-2584396279

Fax: +00-86-2584396279

E-mail: jfchen@njau.edu.cn

Content:

Figs. S1–S4

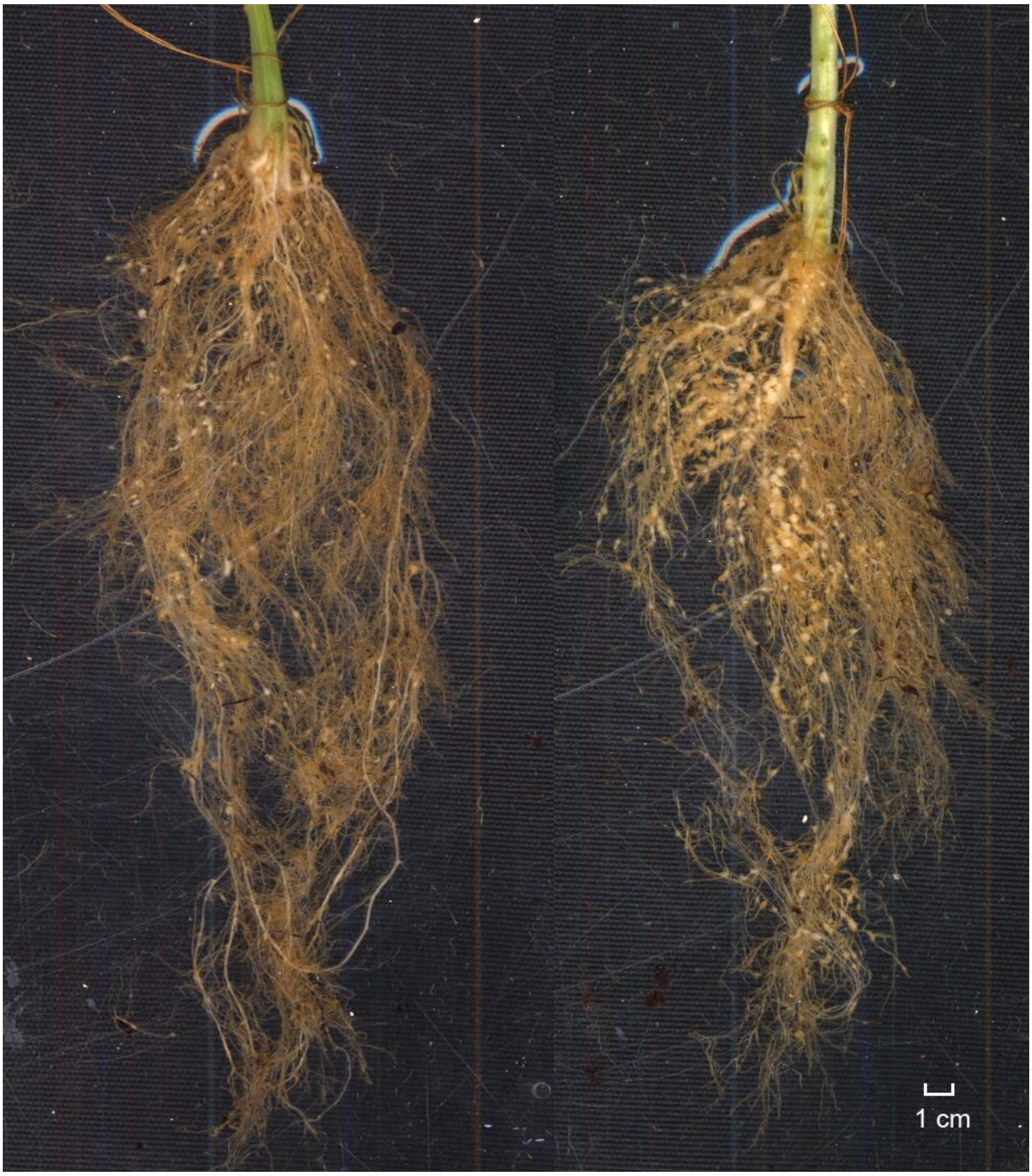

Figure S1 Root symptoms of Introgression line IL10-1 (left) and C. *sativus* var. CC3 (right) after 45 days inoculation with *Meloidogyne incognita*

Note: Bar=1.0 cm

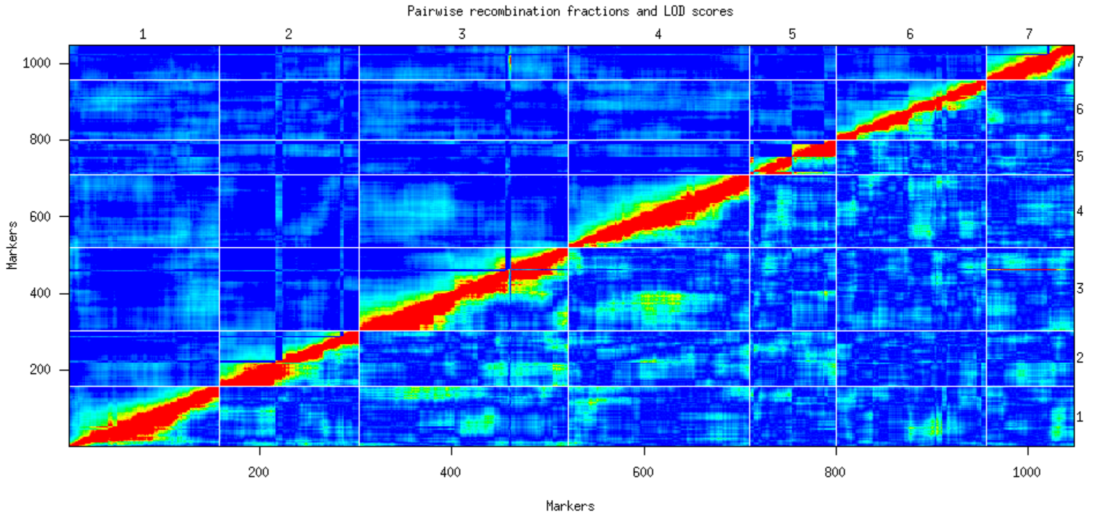

Figure S2 The heat-map of pairwise linkage markers analysis

Plot of estimated recombination fraction (upper-left triangle) and LOD scores (lower-right triangle) for all pairs of markers. Red indicates linked (large LOD score or small recombination fraction) and blue indicates not linked (small LOD score or large recombination fraction)

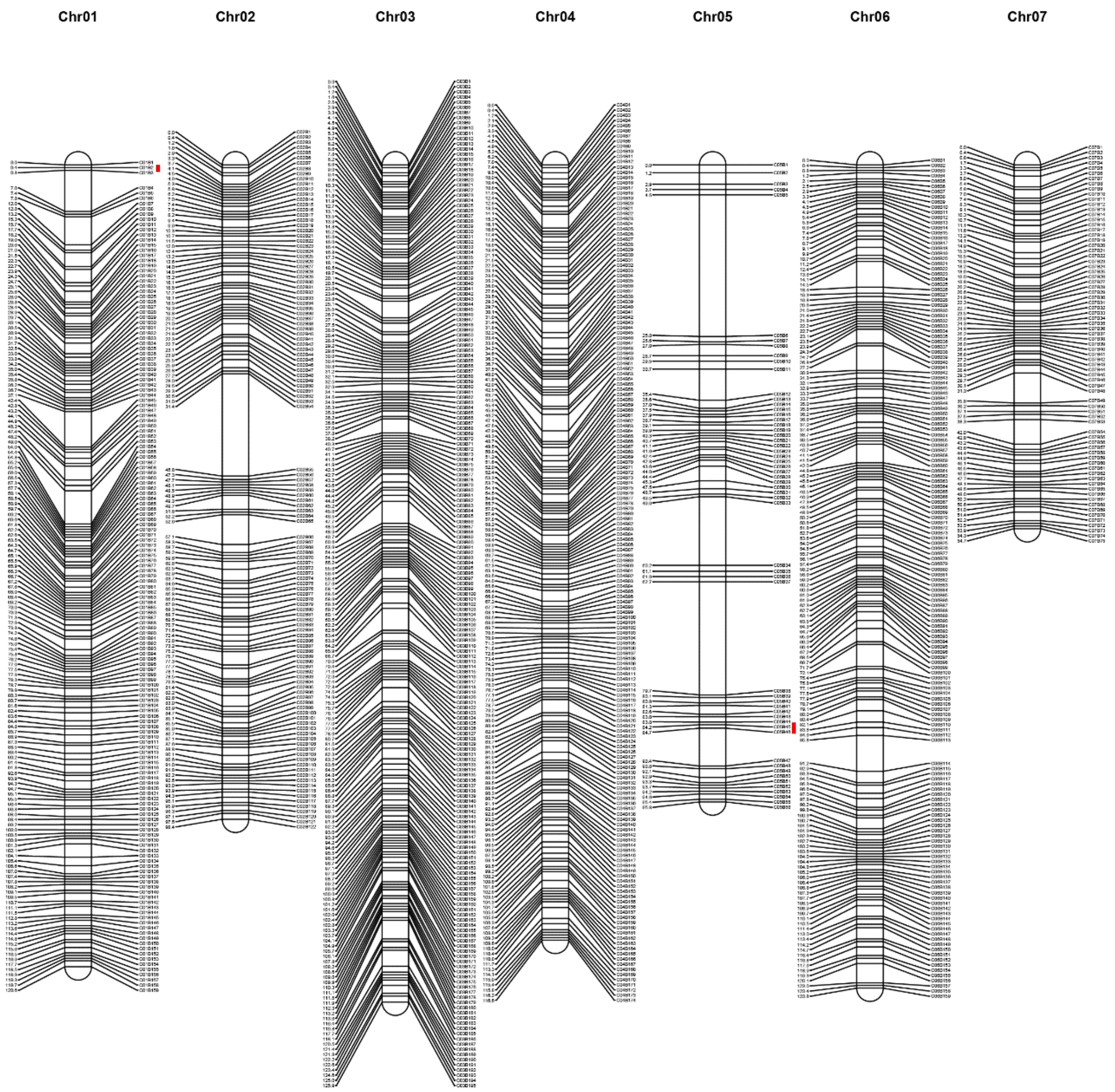

Figure S3 Genetic linkage Bin-map based on the RIL population derived from the cross IL10-1×CC3

Note: Bin markers are named as CnBm, n represents for chromosome number, m represents for Bin number. The red vertical lines mark the locations of QTL.

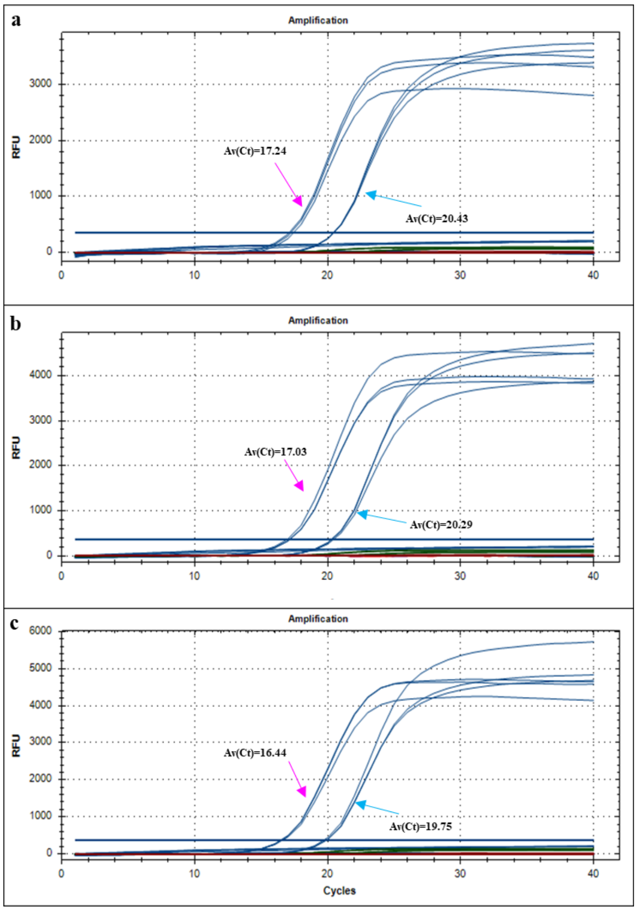

Figure S4 PCR efficiency was calculated for each target from the raw fluorescence data taken using a LightCycler 480 Real-Time PCR System.

a, **b**, **c** show the efficiency of different dulition rates (diluted 10:1) using cDNA of IL10-1 from 1, 2, 3 days post inoculation, which illustrating the efficiency is about 100%. Arrows in blue present cDNA diluted 10 times, and arrows in pink present cDNA without diluted.

Table S1 The Primers designed for RT-PCR analysis of The Four Candidate Genes to RKN

Gene ID	Primer sequences-Forward 5'--------'3	Primer sequences-Reverse 5'--------'3	Product Length (bp)	Product GC %
EF1a	ACTGTGCTGTCCTCATTATTG	AGGGTGAAAGCAAGAAGAGC	98	46.4
Csa5M608240.1	GGTGTGAATTTGAAAGCTAACG	ACACAACCGAATTTAGTGGTTG	132	26.5
Csa5M610420.1	CGTCATGTGAATCTGAGGAGTA	GCATTAAGGAAACAGGCAATGA	202	38.1
Csa5M623410.1	CACCATCCAACGCAACTGACACT	TGGAGATGAACCCAAGTTAGGCG	122	54.9
Csa5M610370.1	TAACTTACAAATTCCGGCCA	AATGAAGGGTAGGGAACAGA	159	42.5

Table S2 Gall index of the F2:3 and F2:6 population, derived from IL10-1×CC3, of four different seasons in different experimental bases

Indlabel	$F_{2:3}$-11Spring	$F_{2:3}$-12Spring	$F_{2:6}$-14Autumn	$F_{2:6}$-15Spring	Indlabel	$F_{2:3}$-11Spring	$F_{2:3}$-12Spring	$F_{2:6}$-14Autumn	$F_{2:6}$-15Spring
**1_2**	3	2.8	4	2.5	**29_1**	3.67	3.6	2.65	3.33
**2_1**	3.33	2.67	4	2	**30_1**	2.5	2.33	4.65	3
**3_2**	2.5	1.6	2.65	1	**31_1**	3	3.33	5	3.67
**4_2**	3.33	2.5	4	1.67	**32_2**	2	2.6	4.35	3
**5_2**	3	2.6	3.65	2	**33_1**	4.33	3.5	4.35	2.67
**7_1**	2.5	2.83	4.5	3	**34_1**	3	3.5	3.65	4
**8_3**	2.67	3.17	4	3.67	**35_2**	3	3.2	4.65	3.33
**9_1**	3.33	3.17	2	3	**36_3**	3	3	4	3
**10_3**	4.5	3	2.65	1.5	**37_1**	4	3.67	4	3.33
**11_2**	3.33	3.4	3.35	3.5	**38_2**	3	3.2	4.5	3.5
**12_3**	3.67	3.5	3.35	3	**39_2**	3.67	3.8	2.65	4
**13_1**	3.67	3.5	4.65	3.33	**40_2**	2.67	2.8	1.65	3
**14_2**	3.67	3.6	2.65	3.5	**42_3**	3	2	3.5	1
**15_1**	3.67	3.33	4	3	**43_2**	2.5	2.2	4.35	3.33
**17_1**	3.33	3.67	3	4	**44_2**	3.33	4.6	3.35	4.33
**18_1**	2.5	2.4	2.5	2.33	**45_1**	3	3	3.5	3
**19_1**	3.67	3	3	2.33	**46_1**	3.67	3.17	4	2.67
**20_1**	4	4	5	4	**47_1**	3	2.5	4.65	2
**21_1**	4.5	4.2	2.35	4	**48_2**	3.67	3.83	3.35	4
**22_1**	3.67	3.33	1.65	3	**49_1**	3.67	3.33	2	3
**23_1**	3	3	3.35	3	**50_3**	3.67	3.33	3.65	3
**24_1**	3.67	2.83	2.35	2	**51_1**	4.33	3.17	5	2
**25_1**	3	3	1.65	3	**52_3**	2.5	2.33	3.65	2
**26_1**	3	3.17	2.5	3.33	**54_1**	3	2	3.35	1
**27_1**	3	3.33	4.5	3.67	**55_1**	3.5	3.4	3.35	3.33
**28_2**	2.67	2.5	3	2.33	**56_2**	3.33	2.17	2.15	1

continued to Table S2

Indlabel	$F_{2:3}$-11Spring	$F_{2:3}$-12Spring	$F_{2:6}$-14Autumn	$F_{2:6}$-15Spring	Indlabel	$F_{2:3}$-11Spring	$F_{2:3}$-12Spring	$F_{2:6}$-14Autumn	$F_{2:6}$-15Spring
**57_1**	3.33	2.4	2.65	1	80_2	3.00	3.60	2.35	4.50
**58_2**	2.33	1.83	4	1.33	81_3	3.33	2.57	4.35	2.00
**59_1**	3.33	2.8	3	2	82_2	2.67	2.83	1.65	4.00
**60_3**	2.33	2.4	2.5	2.5	83_3	3.50	3.20	3.25	3.00
61_2	3.33	2.33	3	1.33	84_1	4.00	3.00	3.65	2.00
62_1	4	2.6	3.65	1.67	85_1	3.67	3.33	1.65	3
63_2	3	2.67	2	2.33	86_3	4	3.67	3.65	3.33
64_3	4.33	3.33	5	2.33	87_1	3.67	3.67	2.65	3.67
65_2	3.33	2.83	3.5	2.33	89_2	3.67	3.33	1.65	3
66_1	2.5	2.6	3.65	2.67	90_1	3	2.4	2.65	2
67_3	3.33	3.4	3.65	3.5	91_1	3.5	3	2	2.67
68_1	3	2.75	3.35	2	92_3	4	3.6	2.35	3.33
69_1	4.33	3.33	0	3.5	93_2	3	3.2	1.35	3.33
70_1	3.33	3.5	2.35	3.67	94_2	2.33	2.67	2.65	3
71_2	3.67	3.6	4.65	3.5	95_1	5	2.5	4.35	1.67
72_1	3.5	3	4.35	2.67	96_1	2.67	2.17	2.35	1.67
73_1	4	3.33	4.65	2.67	97_1	2.33	2.5	3.65	2.67
74_1	4.5	4	4.35	3.67	98_2	2.67	2.83	3	3
75_1	3	3.2	2.65	3.5	99_3	3	3.33	3	3.67
76_2	2.33	2.17	2	2	100_1	3	2.2	2.65	1.67
77_1	3	2.83	1.65	2.67	101_1	2.5	3	3	3.33
78_1	3	2.5	1	2	102_2	3.33	2.5	4	1.67
79_2	5	4.8	1.35	4.67	103_3	3	2.75	1	2.5

continued to Table S2

Indlabel	$F_{2:3}$-11Spring	$F_{2:3}$-12Spring	$F_{2:6}$-14Autumn	$F_{2:6}$-15Spring	Indlabel	$F_{2:3}$-11Spring	$F_{2:3}$-12Spring	$F_{2:6}$-14Autumn	$F_{2:6}$-15Spring
104_2	3	2.17	1.35	1.33	117_1	3.33	2.83	3	2.33
105_1	3.5	2.2	2	1.33	118_1	3.5	4	4	4.33
106_3	3.5	3.25	2.65	3	119_2	3.33	3.83	3.65	4.33
107_2	3	2.5	2	2	120_1	3.67	3.17	1	2.67
108_3	3.67	3.17	3	2.67	121_3	3.5	3.6	3.35	3.67
110_1	3.67	2.67	3.35	1.67	122_3	3	2.33	2.65	2.67
111_1	3.67	2.33	4	1	123_2	3.5	2.8	3	3.67
112_1	3.67	2.5	3	1.33	124_2	3	2.6	4.65	2.33
113_3	3.5	3	3	3.67	125_1	4	3.6	4.35	3.33
114_2	3	2.5	3.35	2	126_3	4	3	1.65	3.33
115_3	4	3.4	2.65	3	127_2	3.33	3.67	3.65	4
116_2	3	3	3.35	0	128_3	4	3.4	3.35	2.5

Table S3 The sequence information of The Four Candidate Genes to RKN

Gene ID	DNA sequence	protein sequence	position of mutations	mutation type
Csa5M608240.1	ATGAGGAAGCTGTTTATTGTTATGTTCTTTGTCTGTTTGGGAAGTTGGAGAAGCTATGCACAAGACAGTGGAGAAGCTCCAATGGAGAA<br>AACAGAGATGGATTCTCTGTTCTCTTCTATTCAAGGCTTTGTGGGTAATTGGTGGAATGGCTCAGATTTATACCCAGATCCCTGTGGATGG<br>ACGCCAATACAGGGGGTTTCTTGTGATATATTTGATGGGCTTTGGTATGTCACTTCACTAAACATTGGACCAACTCATGACAATTCTCTAG<br>CTTGTTCCCCAAATGCTCATTTCAGACAGCAATTGTTTCAACTCAAACACCTTAAAACTCTGTCCTTCTTTAACTGTTTCGTCTCAACAA<br>AAGGGAAGAATTCTGTTTCACTCCCAACTGGAGAATGGCTTAAACTCGCCGGAACTTTAGAATCTCTTGAGTTCAGATCAAACTCCGCA<br>CTTTCCGGACAAATTCCTGCCAGTTTAGGGACTTTGTCAAAGCTGCAATCTTTGGTTCTGTTACAGAACGGATTCGTCGGTGAAATACCA<br>GAAAGTTTTGGCGATTTGATCGAATTGAAACGATTGGTTCTTGCTGGGAACTCACTCACTGGGCCAATCACTCAAAATCTAGGCAAGTT<br>AAGCGAACTACTGATTTTGGATTTGAGTAGAAATTCGTTGTCGGGTTCTTTACCATTAAGTTTGGGAAATTTGACGGCCCTTTTGAAGCT<br>TGATCTAAGTGAGAACAAATTGAGTGGAATTTTGCCTAGTGAATTAGGCAATATGGGAAATTTGACGCTTTTGGACTTAAGCAACAATAG<br>CTTCTACGGTGGATTGAGACAGTCGTTTGAGAAGATGAGTTCCTTAGAAGAAATTATCTTATCCAGCAACCCAATTGGGGGAGAACTCA<br>ATACCATAAACTGGAAAAATCTACAAAA[T->A]TTAGCGATTTTGGATCTTTCCTACATGGGTCTGTATGGGGAAATTCCAGATTCATTATC<br>TGAATTGAAAAGTCTACGGTTTTTGGGACTTAGCCACAACAATCTAACAGGAACCCCCTCTCCAAAACTTGCAAACCTGCCATTTGTTA<br>GTGCAATTTACTTGTTTGGAAACAATTTGTCTGGTGACCTTAAATTCTCCCAACAGTTTTACGGGAAAATGGGAAGGAGATTTGGGGCAT<br>GGGACAATCCTAATCTGTGTTACCCCATTGGAACGTTGGCGGCAAAAAATGCTCCATTTGGTGTGAAGCCATGTGAGGAAGAAAAAGA<br>AGAAGAAGTGGTGAAATTGATGAAGAAACCCATCTCCAAGGCGAGTTTTGATACAAACAATTGGAATTTTGATGTTTCAAAGGGCTATT<br>CTCTAATTGGAAATGAAGAATTTTGGTTGAAATTTGTGGGGAACACATTAACGATGGTTCTGTTAATCAATATGCTTTTATGA	MRKLFIVMFFVCLGSWRSYAQDSGEAPM<br>EKTEMDSLFSSIQGFVGNWWNGSDLYPD<br>PCGWTPIQGVSCDIFDGLWYVTSLNIGPT<br>HDNSLACSPNAHFRQQLFQLKHLKTLSFF<br>NCFVSTKGKNSVSLPTGEWLKLAGTLESL<br>EFRSNSALSGQIPASLGTLSKLQSLVLLQN<br>GFVGEIPESFGDLIELKRLVLAGNSLTGPIT<br>QNLGKLSELLILDLSRNSLSGSLPLSLGNL<br>TALLKLDLSENKLSGILPSELGNMGNLTLL<br>DLSNNSFYGGLRQSFEKMSSLEEIILSSNPI<br>GGELNTINWKNLQ[N->K]LAILDLSYMGL<br>YGEIPDSLSELKSLRFLGLSHNNLTGTPSP<br>KLANLPFVSAIYLFGNNLSGDLKFSQQFY<br>GKMGRRFGAWDNPNLCYPIGTLAAKNAP<br>FGVKPCEEEKEEEVVKLMKKPISKASFDT<br>NNWNFDVSKGYSLIGNEEFWLKFVGNTL<br>TMVLLINMLL*	23326853	T->A
Csa5M610420.1	ATGGCAAAAATTCCTTCAGTATCCTTTTTTTTTCTCCTCTGCAACTTATTATGTCTTTCTATGGCTTCTTTGAACAATGAAGGCAATGCCCT<br>TTTATCATTCAAGCAATCCATTACTGAAGACCCGGAGGGATGTCTTAGCAACTGGAATTCTTCAGATGAAACCCCATGTAGTTGGAATGG<br>AGTTACATGCAAAGATCTAAGAGTTGTTTCTCTTAGTATTCCTAGGAAGAAACTTAATGGGGTTCTTTCTTCTTCTCTAGGGTTTCTGTCT<br>GAGCTTCGTCATGTGAATCTGAGGAGTAATAAACTCCATGGAACTTTGCCAGTGGAGCTTTTTCAAGCTAATGGAATTCAAAGTTTGGTG<br>CTTTATGGAAATTCCTTCACTGGGTCTGTTCCAAATGAGATTGGTAAGCTAAAAAACCTTCAAATCTTTGATTTGTCACAGAATTTCCTTA<br>ATGGGTCATTGCCTGTTTCCTTAATGCAATGTACAAGGTTGAGAATTCTTGATCTTAGTCAGAATAATTTTACTAATTCTCTGCCTAGTGGG<br>TTTGGTTCCAGTTTGAATTTCTTGGAAACGTTGGATCTTTCTTATAATAAGTTTAATGGTTCAATTCCAATGGATATTGGTAATTTGTCTAG<br>CTTACAAGGCACTGTTGATTTCTCACATAATTTGTTTTCTGGCTCTATCCCACCCAGCCTGGGCAACCTTCCTGAAAAGGTTTATATTGAT<br>CTTACTTATAATAATTTGAGTGGTTCAATACCCCAAAATGGTGCTCTTATGAACAGAGGACCAACTGCTTTTATTGGGAATCCTGGCCTTT<br>GTGGACCGCCGTTGAAGAACCCATGTTCTTCCGAAACTCCGGGTGCGAGTTCTCCGTCCTCATTCCCTTTTTTCCCGGACAATTATCCGC<br>CTGGGAGTTCTGAGGGTAATGGTCATAAGTTTGACAAAGGTGGGTTGAGTAGGAGCACTTTAGTTGCAATAATCATAGGCGACATTGTA<br>GGTATTTGCCTCATCGGTTTGCTGTTTTCGTATTGCTATTCGAGGTTTTGCACTCATAGAAATGGGAAGAAGGCAGACCAATCTAGTTATG<br>GATTTGAGAAGGGTGAGAAAGGAAGAAAGGATTGTCTCTGCTTTCAAAAGAGTGAATCTGAGAACGTTTCAGAGCATATCGAGCAGTT<br>TGATTTGGTACCACTGGATTCACAAGTGACATTTGATCTTGACGAACTTCTCAAGGCATCAGCATTTGTTTTGGGAAAAAGTGGTATCGG<br>GATCGTGTATAAAGTCGTGCTTGAAGATGGACTCACTTTAGCTGTGAGAAGATTGGGTGAAGGTGGTTCTCAAAGATTAAAAGAATTTC<br>AAACTGAAGTTGAAGCAATTGGGAGGTTGAGGCATCCAAATGTTGTTAGCCTTAGAGCATATTATTGGTCTGTTGATGAGAAACTGCTTA<br>TTTACGATTACATCCCAAATGGGAACCTTGCATCTGCCGTTCATGGGAAGCCCGGAACAACGTCGTTTACACCTCTACCATGGTCTGTTC<br>GGTTTGGAATCATGA[T->C]AGGCATTGCAAAAGGGTTGGTTTACTTGCACGAGTATAGCCCCAAAAAGTACGTTCATGGTAATTTGAAA<br>ACAAACAACATACTTCTTGGACACGACATGACACCAAAAATTTCTAATTTCGGGCTAGCCCGCCTTGTTAACATTGCTGGAGGATCACC<br>AACCGTGCAATCGAGCCATATAGCAGAGGAAAAATCACAAGAGAAACAACTGAAGTCTGCCACCTCTGAAGCTAGTACATTCAGTTCT<br>AGTATGAGTACTTACTACCAAGCCCCTGAAGCGTTGAAAGTCGTGAAGCCATCACAGAAATGGGACGTTTACTCATATGGTGTGATATTA<br>CTCGAAATGATCACCGGAAGATTGCCCATAGTTCAAGTGGGAACGTCAGAAATGGATCTTGTTCAATGGATTCAACTCTGCATTGAAGA<br>AAAGAAGCCGCTTTCAGACGTAATAGACCCGTCATTGGCTCCAGACGACGATGCTGACGAGGAGATCATAGCGGTTTTGAAGATAGCAT<br>TAGCTTGTGTTCAAAATAATCCAGAAAGAAGACCTGCAATGAGGCATGTTTGTGATGCTTTGGGCAAGTTGGCTGTCACTCCAAACTAA	MAKIPSVSFFFLLCNLLCLSMASLNNEGN<br>ALLSFKQSITEDPEGCLSNWNSSDETPCS<br>WNGVTCKDLRVVSLSIPRKKLNGVLSSSL<br>GFLSELRHVNLRSNKLHGTLPVELFQANG<br>IQSLVLYGNSFTGSVPNEIGKLKNLQIFDL<br>SQNFLNGSLPVSLMQCTRLRILDLSQNNF<br>TNSLPSGFGSSLNFLETLDLSYNKFNGSIP<br>MDIGNLSSLQGTVDFSHNLFSGSIPPSLGN<br>LPEKVYIDLTYNNLSGSIPQNGALMNRGP<br>TAFIGNPGLCGPPLKNPCSSETPGASSPSSF<br>PFFPDNYPPGSSEGNGHKFDKGGLSRSTL<br>VAIIIGDIVGICLIGLLFSYCYSRFCTHRNG<br>KKADQSSYGFEKGEKGRKDCLCFQKSES<br>ENVSEHIEQFDLVPLDSQVTFDLDELLKA<br>SAFVLGKSGIGIVYKVVLEDGLTLAVRRL<br>GEGGSQRLKEFQTEVEAIGRLRHPNVVSL<br>RAYYWSVDEKLLIYDYIPNGNLASAVHG<br>KPGTTSFTPLPWSVRFGIM[I->T]GIAKGLV<br>YLHEYSPKKYVHGNLKTNNILLGHDMTP<br>KISNFGLARLVNIAGGSPTVQSSHIAEEKS<br>QEKQLKSATSEASTFSSSMSTYYQAPEAL<br>KVVKPSQKWDVYSYGVILLEMITGRLPIV<br>QVGTSEMDLVQWIQLCIEEKKPLSDVIDP<br>SLAPDDDADEEIIAVLKIALACVQNNPERR<br>PAMRHVCDALGKLAVTPN*	23758300	T->C

continued to Table S3

Csa5M623410.1	ATGCATCTCCTCCGCCACCATCCAACACCACTGATGCTCCACC[G->T]CCGCCGCCGCCATCCAACGCCACCGACGCTCCACCTCCACC<br>GCCACCATCCAACGCCACTGACGCTCCACCTCCACCGCCACCATCCAGCGCCACTGACGCTCCGCCTCCACCGCCACCATCCAATGCC<br>ACCGACACTCCGCCTCCACCGCCACCATCCAGCACCACTGACGCTCCGCCTCCACCGTCACCATCCAACGCAACTGACACTCCCCCA<br>CCCCCATCCAATACAACCAACTCCCCACCACCACCACCTAATTCCACTGTTAACTTACCACCTGCACCGCCGCCTAACTTGGGTTCATC<br>TCCACCACCGTCGCCAACGCCTAATGGAGCATTGA	MHLLRHHPTPLMLH[R->L]RRRHPTPPT<br>LHLHRHHPTPLTLHLHRHHPAPLTLRLH<br>RHHPMPPTLRLHRHHPAPLTLRLHRHH<br>PTQLTLPHPHPIQPTPHHHHLIPLLTYHL<br>HRRLTWVHLHHRRQRLMEH*	24777458	G->T
Csa5M610370.1	ATGAGTGATATCAAATTCCGTTTCGCTCTGTTTTTTGTTCTCTCTCATATAGTGGCACTTTCGTTGGCTTGGAATGATACCAGATACGTTC<br>ATCCAATTTGCACGCCCTTCGAATGTGGAAATCTGGGCCTCATAGGATTCCCTTTTAACAATATGTCGCTGACTGATTGTGGATTTTACA<br>CAGTTAAAAACTGCTCGGGACAGCCTAAAATCCAGCTCAATCGTGGAAAAGAATTATGGTTTGATGTTACTACCATTTCTCAGGCCAAT<br>GTCATCCATATCAATGACCAAGAACTTCGGAAGCGAATAAATGCTCGCAATTGCACTATTCTCGATGATCTAGCTCTTCCAATATCTTCT<br>TTGTCTTCTTTATCCACAGATAATAATCTTACTATGTATTATTGTACAGAAAAACCTAAAGACGCTTTACCTCTTTTCATCAGTTCATTTAA<br>TTGCCCTGATTATTACACATACCTCAACACTAGTGCTTCACCCAACTGTCTCACTTCCAAGTCTAAATTTGTTGTTCCAGTTCGCCCCGT<br>TGGTCCCAATAACTCAGTTCTTGAGTTTACTTCTAATTTCCAACTTCAAGTAACCATATCCGTTTCTTGTCAAGGATGTTTTCATAGAGG<br>AGGCCGATGCTCGGATACCCAAGGATACTTCGTTTGTGAAGGTGAAAACACAAAAAGTAGAAAAGCAAAACCTGATATTTGGGGGAT<br>TGCACTAGGGAGCTTTACTACGGTAATATCCATCATCATCATCTTCATCATCATCTACAACAAAACAAAAGGCATGTCAAACAAAAACA<br>AAATTAATGAAGTCATTGGAAGATATTCAACCCACACTCCCAAGCGATACACTCACTCAAAGCTAAAGAAAATCACAAACTCCTTCAA<br>GAACAAGCTTGGCCAAGGAGGATTCAGCTCTGTCTACAAAGGGAAATTACCCGATGGCCGTGAAGTGGCAGTGAAACTACTGAATGA<br>ATGCACAGAAAACGATGAAGACTTCATGAATGAAGTCGTTAGCATCACAAGAACTTCACACGTAAACATAGTCAAGCTTTTTGGTTTC<br>TGCTACGAACAGGGAAAAAGAGCATTGGTTTATGAGTACATGTCTAAAGGGTCATTAGATAAGTACATTTTTCAAAG[A->T]GGGCAAC<br>AAAAAGAAAATGAACAAGTTTTGGATTGGAAAATGTTGAATAGCATAGTTATGGGCGTAGCACGAGGATTAGAGTACCTGCATCGAGG<br>CTGCAACACGAGGATTTTTCATTTTGACATCAAACCACACAACATTCTTTTGGACGATGACTTCTGTCCCAAAATCTCGGATTTCGGGC<br>TTGCCAAGCAATGCAAGGCTAGAGAGAGTCACGTGTCGATGACGGGTGCAAAGGGGACTGCGGGATTTATGGCGCCAGAAGTAATGT<br>ATAGAAACTTTGGCAAAGTTTCGCATAAGTCTGATGTTTATAGTTTTGGAATGTTGGTTCTTGAGATGGTGGGTGAAAGAAAGAGACC<br>AGATGAATTAAGAGTTGGGGAGATCAGCGAAGAAGAGTACTTCCCGGATTGGATATATAAGGATTTGGAAAAAAGTGGAATAGATAGA<br>GCGCATTGGTGGGGAAGAACGGAGGAAGAACAAGAAATGGCAAGAAAAAATGATCATTGTCGGACTGTGTTGTATACAGACGTTGCC<br>GGAGGACAGGCCATCAATAAGCGATGTGGTTGAGATGTTGGAAGGGAGTGTTGATAACTTACAAATTCCGGCCAAGCCGACCTTATTT<br>GGACCTCCTGCTATCTTTCTCCACCCTCCTCCTTCTTCTTCTTCTTCATCCTTTCGTGCAACCTGCAGTGACTACACCCAAGTTTTTTCC<br>TTCCCCTCCCTTCTCTCTGTTCCCTACCCTTCATTTTCTGCCATTAAAACAAACAATCTCCAGGAAATATGCTTTACCCAGAATGTGACA<br>CTGGGGGAGTGA	MSDIKFRFALFFVLSHIVALSLAWNDTR<br>YVHPICTPFECGNLGLIGFPFNNMSLTD<br>CGFYTVKNCSGQPKIQLNRGKELWFD<br>VTTISQANVIHINDQELRKRINARNCTIL<br>DDLALPISSLSSLSTDNNLTMYYCTEKP<br>KDALPLFISSFNCPDYYTYLNTSASPNC<br>LTSKSKFVVPVRPVGPNNSVLEFTSNFQ<br>LQVTISVSCQGCFHRGGRCSDTQGYFV<br>CEGENTKSRKAKPDIWGIALGSFTTVIS<br>IIIIFIIIYNKTKGMSNKNKINEVIGRYST<br>HTPKRYTHSKLKKITNSFKNKLGQGGF<br>SSVYKGKLPDGREVAVKLLNECTENDE<br>DFMNEVVSITRTSHVNIVKLFGFCYEQ<br>GKRALVYEYMSKGSLDKYIFQ[R->S]G<br>QQKENEQVLDWKMLNSIVMGVARGLE<br>YLHRGCNTRIFHFDIKPHNILLDDDFCP<br>KISDFGLAKQCKARESHVSMTGAKGT<br>AGFMAPEVMYRNFGKVSHKSDVYSFG<br>MLVLEMVGERKRPDELRVGEISEEEYF<br>PDWIYKDLEKSGIDRAHWWGRTEEEQ<br>EMARKMIIVGLCCIQTLPEDRPSISDVV<br>EMLEGSVDNLQIPAKPTLFGPPAIFLHPP<br>PSSSSSSFRATCSDYTQVFSFPSLLSVPY<br>PSFSAIKTNNLQEICFTQNVTLGE*	23735224	A->T
